# RGB Trichromatic Whiteness Assessment of Bio Analytical Chromatographic Tool Using Fluorescence for Quantitation of Semaglutide: Application to Pharmaceutical Preparations and Spiked Plasma

**DOI:** 10.1007/s10895-024-03954-9

**Published:** 2024-10-28

**Authors:** Mona M. Abdel Moneim, Miranda F. Kamal, Mohamed M. A. Hamdy

**Affiliations:** 1 Department of Pharmaceutical Chemistry, Faculty of Pharmacy, Pharos University in Alexandria, Canal El Mahmoudia Street, Beside Green Plaza Complex 21648, Alexandria, Egypt; 2 Department of Pharmaceutical Analytical Chemistry, Faculty of Pharmacy, Damanhour University, Beheira, Egypt

**Keywords:** Semaglutide, HPLC, Flourescence, Plasma, Green Analytical Chemistry

## Abstract

Semaglutide (SEMG) is one of the most widely used and trending medications to treat type II diabetes and obesity. This work aimed to develop a liquid chromatography with spectroflourimetric detection (HPLC-flourimetry) analysis of SEMG in both its tablet dosage form and plasma. The power of fluorescence detection coupled with HPLC proved its capability as a bioanalytical tool to assay SEMG in plasma samples owing to its simplicity and sensitivity which reached below the C_max_ of SEMG. Separation was done using a C_18_ column with mobile phase of acetonitrile and water acidified with orthophosphoric acid (pH 3.5) (1.41 × 10^–5^ M) in isocratic mode in ratio 57:43 and 1 mL/min flow rate after extraction using protein precipitation. Detection was carried out at λ excitation of 238 nm and λ emission of 416 and 307 nm for SEMG and the internal standard, respectively. Evaluation of greenness of the proposed method was done using AGREE (Analytical GREEnness Metric Approach), ComplexGAPI (Complementary Green Analytical Procedure Index) & the new algorithm RGB 12 model (Red–Green–Blue). They showed that these methods can be a greener alternative with acceptable sensitivity for analysis of SEMG. The developed seven min-assay was validated per ICH as well as FDA bio analytical methods’ guidelines to prove its applicability for routine sample analysis and future pharmacokinetic studies.

**Supplementary Information:**

The online version contains supplementary material available at 10.1007/s10895-024-03954-9.

## Introduction

“Diabesity” (Obesity and Type-2 diabetes) is one of the biggest epidemics worldwide. Thus, there are continuous efforts and focus worldwide on this epidemic regarding its etiology, epidemiology as well as its preventive, control and therapeutic drugs [1, 2].

Balanced and sustainable lifestyle, customized diets, strength training, metabolic boosters and appetite suppressants are globally manipulated in scientific conferences, commercials and OTC (over the counter) markets to be able to manage diabetes as well as achieve the urge of adults and teenagers for weight loss.

“Glucagon-like peptide 1 (GLP-1) agonists” are a new category of type 2 diabetic drugs, that controls blood sugar levels and are recently repurposed as weight loss medications. GLP-1, agonists are taken by a subcutaneous shot (injection) daily or weekly. They include; Dulaglutide (Trulicity), Semaglutide (Ozempic, Rybelsus), Liraglutide (Victoza, Saxenda) and Lixisenatide (Adlyxi) [3].

GLP-1, a physiological hormone, promotes glycemic control via several different mechanisms, including: secreting insulin, slowing the gastric emptying, as well as reducing postprandial glucagon secretion. Semaglutide, SEMG, is 94% similar to human GLP-1. Consequently, it stimulates insulin synthesis and reduces glucagon secretion [4].

SEMG was first developed by “Novo Nordisk” and approved by U.S. FDA as anti- diabetic subcutaneous injection in 2017, then as tablet formulation in 2019. Meanwhile, FDA approved it in weight reduction for diabetics, “diabesity”, in 2021. SEMG together with low calorie diet and high physical activity in adults is used to manage obesity in individuals with one of the common weight related health condition as high blood pressure, type 2 diabetes and high cholesterol [5]. Furthermore, SEMG has lately (in March, 2024), gained FDA approval for reducing the risk of serious cardiovascular problems especially for diabetic and obesity patients [6].

Rapid and pervasive promotion in social and pharmaceutical marketing media has led to an inflated demand for the injectable and/or oral SEMG formulations. Thus, chemistry and stability studies of SEMG as well as its possible analytical screening in varying matrices are mandatory for its estimated huge production [7].

**Fig. 1:**
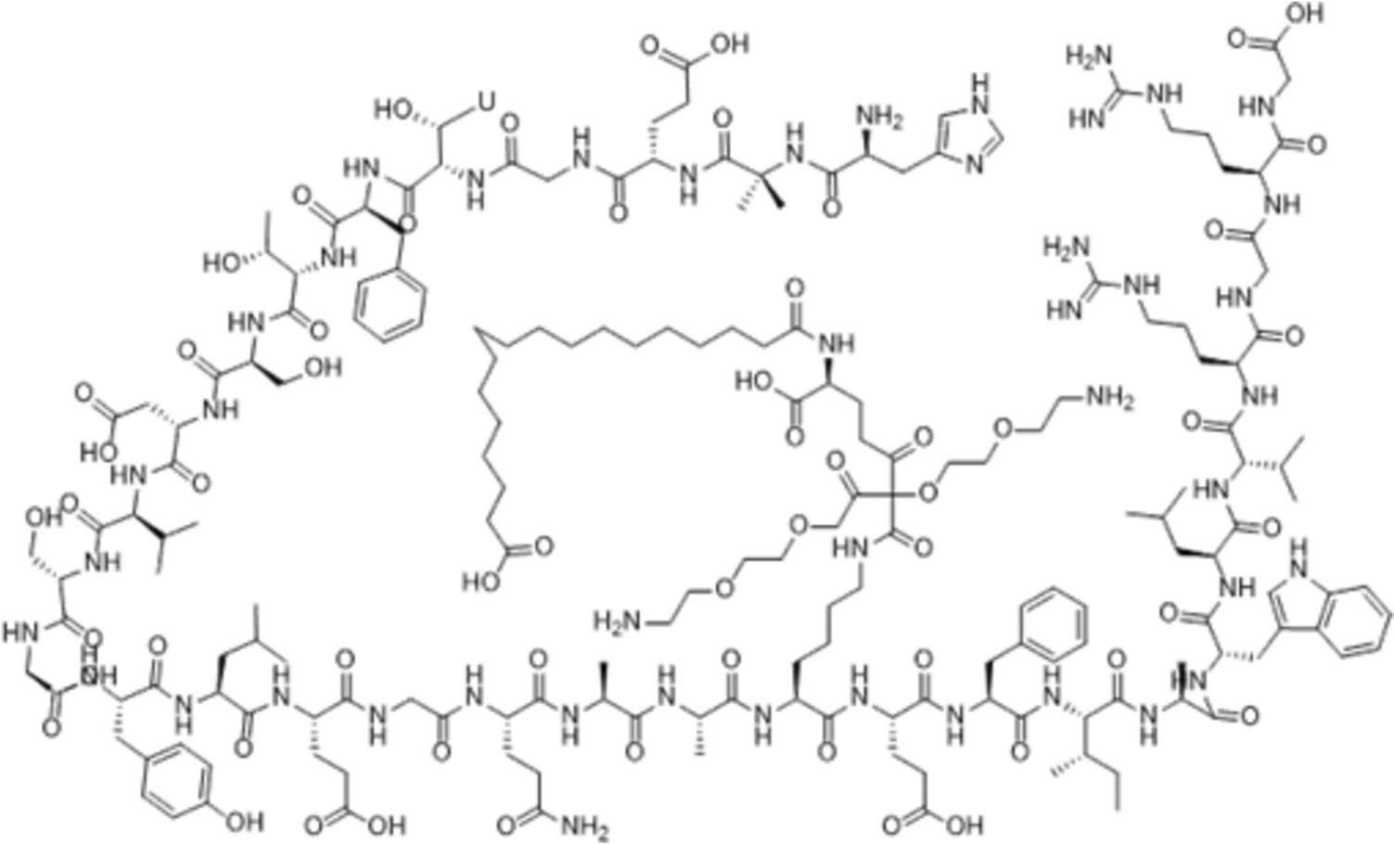
Semaglutide (SEMG) chemical structure

SEMG average molecular weight is 4113.64, chemical formula is C_187_H_291_N_45_O_59_, as shown in Fig. 1. Its C_max_ is 10.9 nmol/L, and it has high binding affinity to plasma albumin (more than 99%), so it has high levels of stability and long half-life of 168 h. Main elimination route of SEMG is the urine, i.e., through the kidneys [8].

For SEMG assay, the literature reveals the technical application of chromatography and spectrometry. HPLC-Mass Spectrometry determination of SEMG for pharmacokinetics and its brain distribution in rats has been lately studied [9]. RP-UPLC for SEMG quantitation in bulk and formulated product [10] as well as stability-indicating LC-DAD [11, 12] and UV spectrometric methods [13] have been overviewed since 2018. Recently, native fluorimetric determination of SEMG, [14] has been reported.

The present study describes the first HPLC-fluorimetrically detected quantification of SEMG as bulk, formulated forms and spiked in human plasma using Chlorzoxazone (CLZ) as internal standard. The fluorimetric properties of SEMG enabled its chromatographic determination in spiked human plasma with acceptable sensitivity and selectivity, without using high cost and highly consuming instruments as HPLC–MS. The method in our study was ICH validated and sensitive, selective and accurate assay results have been obtained.

Since green chemistry and analysis is the outmost goal in the present time, thus developing new green analytical techniques to substitute non-environmentally safe ones is an everlasting target. The proposed fluorimetric based HPLC method was thoroughly assessed for its greenness using different approaches and proved its advantages and environmental sustainability.

## Experimental

### Chemicals and Reagents

SEMG (99%) and CLZ (99.9%) were supplied by Biosynth (UK) and GlaxoSmithKline Co. (Egypt), respectively. Rybelsus® tablets containing 14 mg SEMG (Novo Nordisk A/S, Denmark) were purchased from our local market. Acetonitrile and methanol HPLC-grade (Sigma-Aldrich Chemie GmbH, Switzerland), ortho-phosphoric acid (BDH Laboratory Suppliers, England) and double distilled water were used in our study. Human plasma was supplied from the Alexandria blood bank, Egypt.

### Instrumentation

The HPLC assay was performed using Agilent 1260 device (USA) equipped with fluorescence detector (G1321C). A reversed phase Agilent-C18 (250 × 4.6 mm) column thermostated at ambient temperature (25 °C) has been used with mobile phase of acetonitrile and water acidified with orthophosphoric acid to pH 3.5 in ratio of 57:43 at a flow rate of 1 mL/min. The fluorescence detector was set at λ excitation of 238 nm and λ emission of 416 and 307 nm, for determination of SEMG and CLZ, respectively.

## Methods

### Standard Stock Solutions and Calibration Curves

Stock solutions of SEMG and CLZ (250 µg/mL) were prepared by dissolving 25 mg, of each in 100 mL methanol. The SEMG stock solution was further diluted to prepare a 10 µg/mL (for dosage form analysis) and 100 µg/mL (for spiked plasma analysis) working solution in methanol. CLZ was also further diluted in methanol to a working standard of 10 µg/mL.

#### For Dosage Forms Analysis

The calibration standards were prepared by transferring accurate micro-volumes (10–1000 µL) of SEMG working solution into a set of five 10- mL volumetric flasks and diluted to volume with methanol to obtain a final calibration curve in range of 0.01–1.00 µg/mL. Triplicates of 20-µL injections of each prepared standard were injected in the previously mentioned chromatographic system.

#### For Spiked Plasma Extraction and Analysis

Five appropriate volumes (25–250 µL) of SEMG standard working solution (100 µg/mL) were transferred into a set of five 10 mL volumetric flasks and completed to mark with methanol to achieve a final working concentration range of (0.25–2.5 µg/mL). Five plastic Eppendorfs containing 250 µL plasma were spiked separately with 25 µL of each of the prepared SEMG working solutions (0.25–2.5 mg/mL) and 25 µL of IS solution, from its working solution of 10 µg/mL, followed by 500 µL of acetonitrile for protein precipitation to achieve concentrations in the range of 0.025–0.25 µg/mL plasma for the SEMG with fixed IS concentration of 1 µg/mL plasma in all samples. After vortex mixing (2 min) to ensure complete extraction, centrifugation (15 min, -4 °C & 14,000 rpm) was done to separate the precipitated proteins and achieve a clear supernatant of the extracted SEMG in acetonitrile. The supernatant layer was filtered with a 0.45 µm syringe adapter to ensure a clear solution with no micro particles that may clog the HPLC column or syringe and affect the separation and 20 µL was injected in triplicates in the chromatographic system. (n = 5). Similarly, the Quality control standards, QCs were prepared, 0.025, 0.075, 0.1, and 0.25 µg/mL plasma to be considered the “LLOQ, lower limit of quantification”, “LQC, low QC”, “MQC, mid QC” and “HQC, high QC”, respectively.

### Analysis of SEMG Tablets Dosage Forms

Five Rybelsus® tablets were weighed and powdered. Into a 25 mL volumetric flask, a powder weight equivalent to 25 mg SEMG was transferred using 20 mL methanol. After 15 min sonication, the flask was completed to volume with methanol and filtered. Appropriate dilutions in methanol were made to achieve final concentration ranges for SEMG analysis and the chromatographic procedure was then continued where triplicates of 20-µL injections of each prepared solutions were injected (n = 5).

## Results & Discussion

### Chromatographic Conditions

**Fig. 2:**
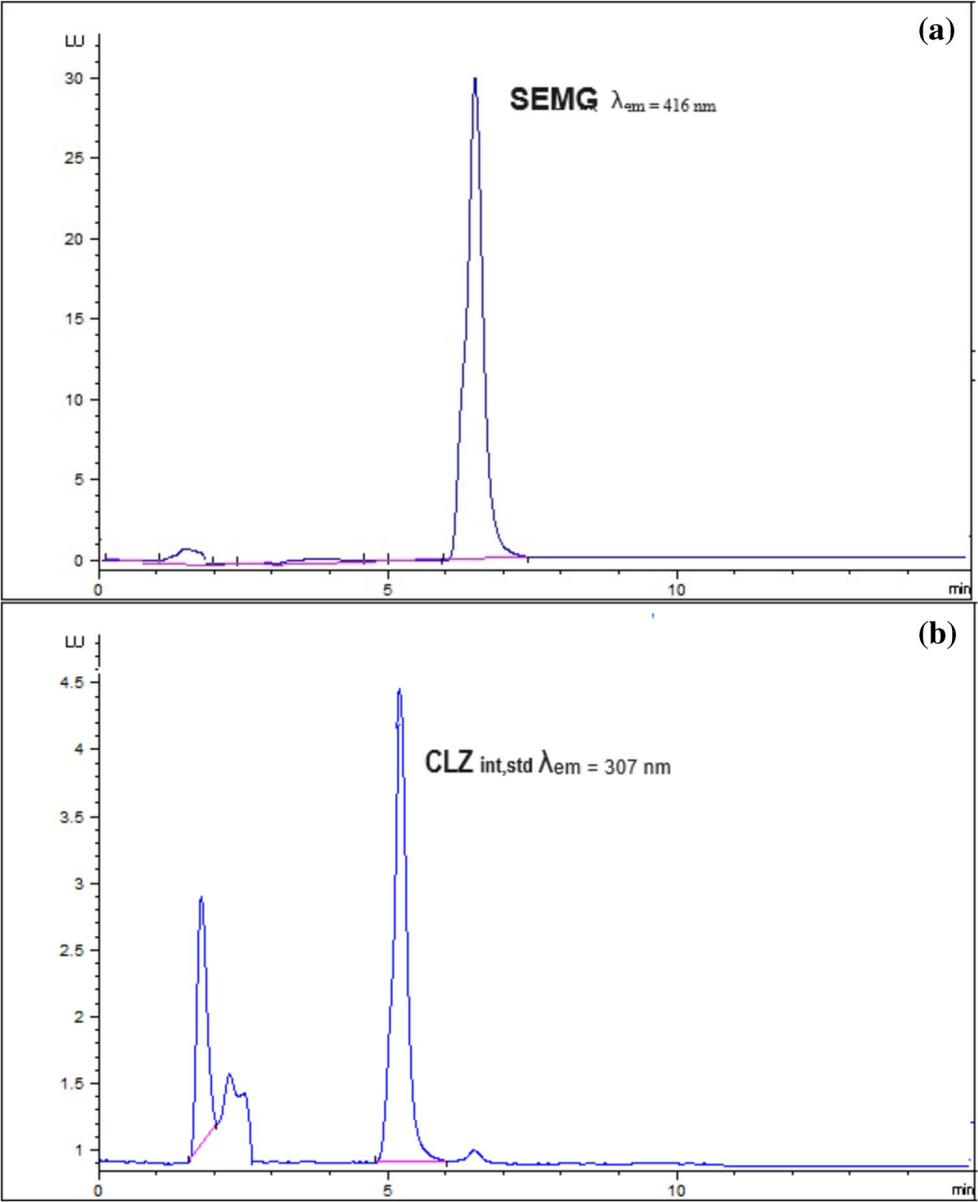
HPLC chromatogram representing (**a**) SEMG in its tablet sample solution at λ_em_ = 416 nm and (**b**) CLZ as an internal standard at λ_em_ = 307 nm

**Fig. 3:**
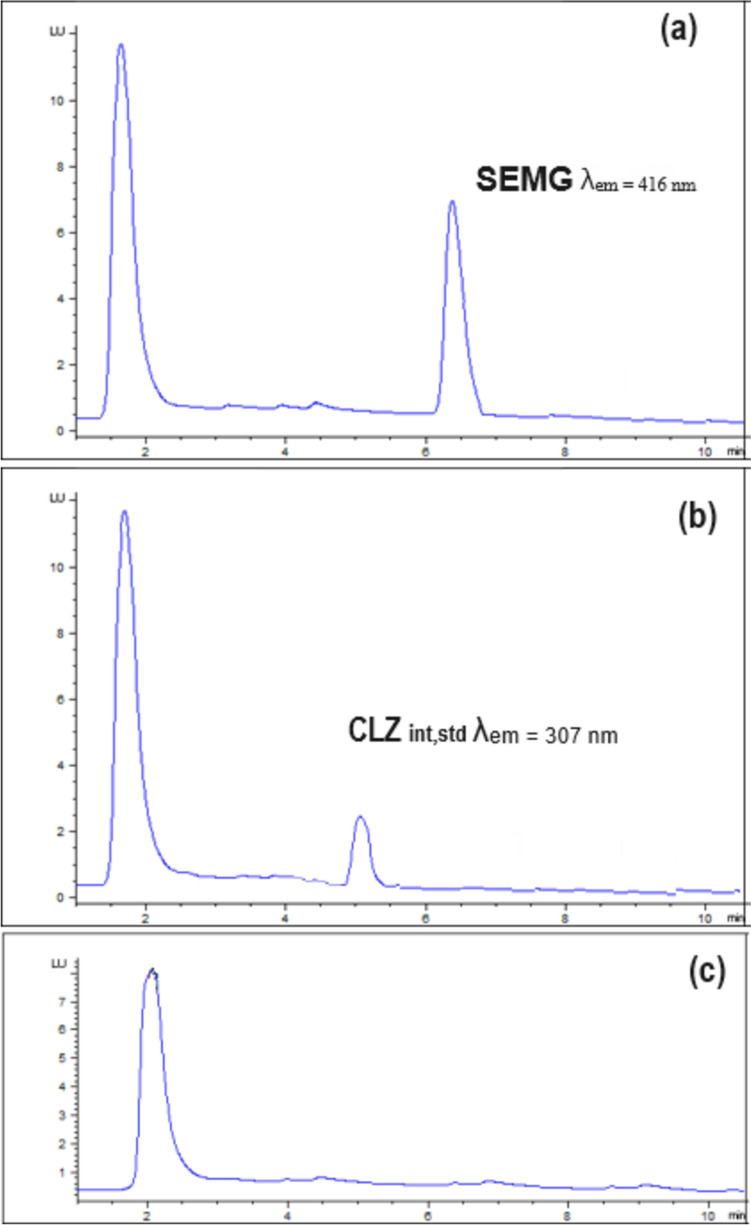
HPLC chromatogram representing plasma sample spiked with LLOQ of SEMG and CLZ as an internal standard (**a**) at λ_em_ = 416 nm and (**b**) at λ_em_ = 307 nm & (**c**) blank plasma

Owing to SEMG native fluorescence, the highly selective chromatographic assay coupled with fluorimetric detection was attempted for SEMG quantitation in plasma. The suggested procedure has achieved the separation and determination of SEMG, in the presence of internal standard of CLZ, in satisfactory resolution. The whole run time has not exceeded 7.5 min; where SEMG peak emerged at Rt of 6.5 ± 0.2 min, at λem 416 nm and CLZ peak emerged at 5.21 ± 0.2 min, at λem 307 nm. (Figs. 2 and 3) Repetitive attempts were carried out to select the optimum analysis conditions, well-separated peaks and a highly selective method in reasonable time using the Agilent (C18, 5 µm, 4.6 × 250 mm) column at room temperature. Different mobile phase composition, proportions, flow rates and pH values of buffer were employed in this study.

After trying both acetonitrile and methanol, acetonitrile was the optimal organic eluent, displaying distinctive LC signal in appropriate retention time for both SEMG and its internal standard. At the beginning, phosphate buffer was used with acetonitrile as an aqueous phase but the SEMG peak was tailed and its symmetry was improved by using water acidified with phosphoric acid instead. The pH effect was also studied from 3 to 5. Optimum pH value of 3.5 produced sharp symmetrical peaks. Thus, mobile phase of acetonitrile: distilled water acidified with phosphoric acid (pH 3.5) was finally tried, in different ratios in isocratic mode. It was observed that the increasing the aqueous phase percentage led to increase in retention time (R_t_ ≥ 10 min) and still the separation between SEMG and CLZ (IS) did not change. Thus, choosing a ratio of 57:43 acetonitrile: aqueous phase was optimum to achieve an appropriate separation in a run time less than 10 min. After optimization of all chromatographic conditions, all system suitability parameters have been assessed and compared to reference values to ensure the validity of the analytical method proposed (Table 1).

**Table 1:** System suitability testing of the chromatographic peaks

Analyte	Retention time (R_t_), min	Capacity factor (k′)	Selectivity (α)	Resolution (R_s_)	Asymmetry factor (A_s_)	Efficiency (plates/m)
SEM	6.50	2.82	––-	––-	1.18	2704.10
CLZ (IS)	5.21	2.06	1.37	2.72	1.11	2144.72

k′ (2 − 10), α > 1, R_s_ > 2, A_s_ (0.8–1.2) and plates.m^−1^ (> 2000). λ_em_

### Sample Extraction

Biological samples preparation and cleanup is a necessity before analysis, to avoid interference from the matrix components. Several procedures are reported for sample clean up including liquid–liquid extraction, solid phase micro- extraction, supercritical fluid extraction and a lot of other techniques. Protein precipitation especially prior to flourimetric analysis is the method of choice for extraction in several reports [15, 16] due to its simplicity and greenness as it requires small amount of organic solvent to precipitate the proteins followed by a simple step of centrifugation and filtration as previously discussed under *“For spiked plasma extraction and analysis”* in Method’s section. Protein precipitation is considered one of the basic extraction techniques, yet achieves the required recoveries with minimal preparation steps and solvent consumption unlike the other mentioned extraction techniques which require special instruments or solvents. Optimization of the protein precipitation procedure was also done by trying different precipitating agents (methanol, acetonitrile or combination of both) and acetonitrile was optimum as it resulted in clearer supernatant as it is a more powerful precipitating agent. In some conditions, protein precipitation technique requires using strong acids or pH adjustments to achieve a complete precipitation for plasma proteins but in the presented work only acetonitrile was enough in a 2:1 ratio (acetonitrile: plasma) to achieve complete protein precipitation and obtain good recoveries. Also the supernatant after filtration was directly injected in the HPLC system achieving the required method sensitivity without the need to evaporate the solvent to minimize the solvent volume and increasing the sensitivity as usually done after protein precipitation step.

### Fluorescence and Internal Standard Measurements

Fluorimetric measurements offers high sensitivity as well as selectivity as usually interfering components do not exhibit native fluorescence properties. This advantage was used in this study, as SEMG has native fluorescent nature which allowed its determination in dosage forms and plasma samples below its reported C_max_ [8]. Thus, the proposed HPLC method could be applied as a bio analytical tool for pharmacokinetics study of this rapidly emerging new drug.

Choosing an internal standard in this study was challenging because it had to be a native fluorescent compound that will elute at an appropriate retention time with respect to SEMG, and yet achieves no interference with the early eluting plasma matrix. Upon scanning the UV spectrum of SEMG in methanol to choose the optimum wavelength of excitation, it showed 3 maximum wavelengths (215, 238 and 290 nm). For detection, the best λ_ex_ was 238 nm as CLZ the chosen internal standard shows λ_ex_ of 244 nm so both drugs will give a high fluorescence intensity at the chosen λ_ex_ (238 nm) but CLZ emission had to be measured at λ_em_ of 307 nm. This was enabled by the capabilities of the fluorescence detector to measure the eluent at different emission wavelengths simultaneously at the same run, so it was set at λ_em_ 416 and 307 nm for SEMG and CLZ, respectively.

### Method Validation

The method in this study was validated according to the International council on Harmonization guidelines [17] as well as the FDA bio analytical method validation guidelines [18] and the results are discussed below.

#### Linearity, Range and Detection (DL) & Quantitation Limits (QL)

Linearity has been assessed to determine the range in which the analyte’s responses (peak areas, or peak areas/IS peak area, for plasma analysis) are linear with the concentrations shown in Table 2. Calibration plots are demonstrated in supplementary file. All regression parameters including the slopes, intercepts, and correlation coefficients (r ≥ 0.9990) together with the standard deviations of intercept (S_a_), slope (S_b_) and residuals (S_y/x_) are demonstrated in Table 2 and within the acceptable limits. Also, DL and QL are calculated as signal to noise ratio 3/1 and 10/1, respectively.

**Table 2:** Parameters of regression for SEMG by the proposed methods

Parameter	Dosage Forms	Spiked Plasma
Linearity range, (µg/mL)	0.01–1.00	0.025–0.25*
QL (µg/mL)	0.01	0.02
DL (µg/mL)	0.003	0.006
Intercept	7.17	1.20
Slope	587.01	35.25
Correlation Coefficient, r	0.9993	0.9992
Standard deviation of intercept (S_a_)	5.74	0.11
Standard deviation of slope (S_b_)	12.37	0.81
Standard deviation of residuals (S_y/x_)	10.18	0.14
F	2250.44	1913.02
Significance F	2.06 × 10^–5^	2.63 × 10^–5^

^*^ µg/mL plasma

Regarding determination of SEMG in plasma, the proposed method offered a LLOQ of 0.025 µg/mL plasma which is lower than the reported C_max_ of SEMG (0.04 µg/mL plasma). Consequently, the proposed method is of suitable sensitivity for the quantitation of SEMG in routine pharmacokinetic studies.

#### Accuracy and Precision

In order to assess the accuracy and precision in case of determination of SEMG in its dosage form, three different concentrations were assayed using the proposed method including the lower limit, upper limit and a mid- concentration. Meanwhile for validation of the bio-chromatographic method in accordance to the FDA guidelines for bioanalytical methods validation, the proposed HPLC method was used to assess the LLOQ, LQC, MQC and HQC. All assays were repeated on the same day six times and on different days as well to study the inter – and intra- day precision. All mean percentage recoveries, relative error values and relative standard deviation are given in Table 3 and show the validity of the method regarding accuracy and precision.

**Table 3:** Precision and accuracy assessment for SEMG determination by the proposed methods

	Intra-day (*n* = 6)		Inter-day (*n* = 6)	
Concentration (µg/mL)	Mean % Recovery ± %RSD	%E_r_	Mean % Recovery ± %RSD	%E_r_
HPLC- Fluorimetric detection *(tablets dosage form)*				
0.01	99.99 ± 0.90	-0.01	98.55 ± 1.00	-1.45
0.2	100.45 ± 1.01	0.45	99.50 ± 1.20	-0.50
1	100.20 ± 0.99	0.20	99.65 ± 1.01	-0.35
HPLC- Fluorimetric detection *(spiked plasma samples)*				
LLOQ	96.97 ± 1.80	-3.03	98.56 ± 1.75	-1.44
LQC	98.50 ± 1.95	-1.50	101.66 ± 1.90	1.66
MQC	98.55 ± 1.88	-1.45	99.35 ± 1.60	-0.65
HQC	101.95 ± 1.50	1.95	100.65 ± 1.99	0.65

#### Recovery Validation

Recoveries of LQC and HQC spiked SEMG plasma samples, were measured by comparing the spiked samples to standard solutions. All extraction recoveries were between 95–105% and recovery of CLZ (IS) was 94%.

#### Robustness

Robustness of the HPLC-FD method was assessed through analysis of SEMG sample solutions with deliberate variation of HPLC parameters. All variations showed no influence on separation and recoveries of SEMG (Table 4).

**Table 4:** Robustness of the proposed HPLC method

Parameters tested	HPLC method (*n* = 3)			
	SEMG *(tablets dosage form)*		SEMG *(spiked plasma samples)*	
	RSD % of peak areas	R_t_ ± SD	RSD % of peak areas	R_t_ ± SD
1) Mobile phase ratio [± 1% organic phase]	0.99	6.49 ± 2.65 × 10^–2^	1.12	6.51 ± 4.09 × 10^–2^
2) Flow rate [1 ± 0.1 mL/min]	0.85	6.50 ± 9.95 × 10^–2^	1.50	6.47 ± 4.55 × 10^–2^
3) Column temperature [25 °C ± 2 °C]	0.88	6.39 ± 9.35 × 10^–2^	0.90	6.48 ± 5.09 × 10^–2^
4) pH of the aqueous phase [3.5 ± 0.2]	1.50	6.50 ± 5.55 × 10^–2^	1.80	6.49 ± 5.35 × 10^–2^
5) λexc (± 2 nm)	0.35	6.51 ± 3.25 × 10^–2^	0.50	6.50 ± 4.55 × 10^–2^

#### Selectivity

**Fig. 4:**
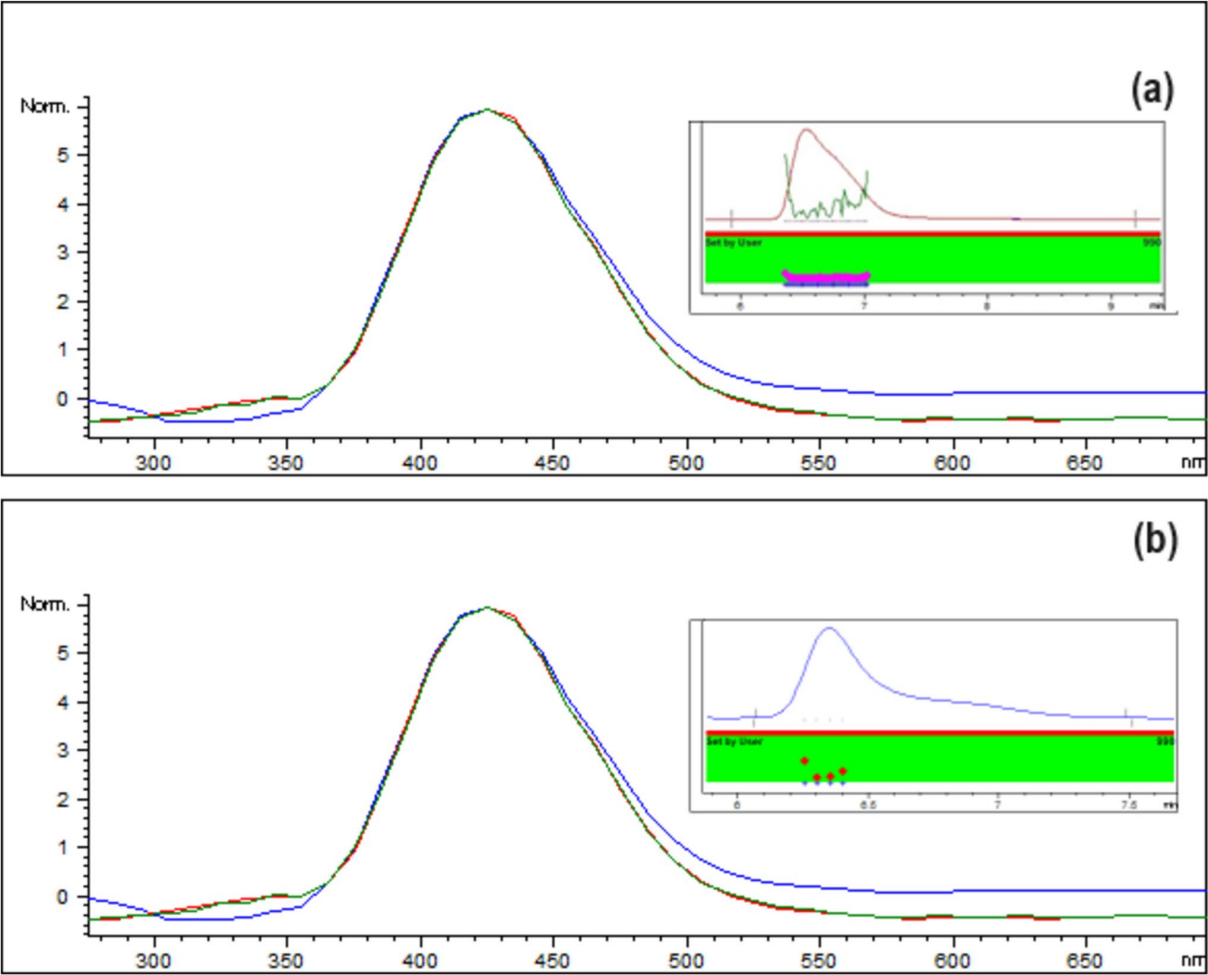
Overlaid emission spectra showing peak purity of SEMG peak (**a**) in tablets sample solution and (**b**) in plasma samples

Selectivity of the HPLC analysis for SEMG using the proposed technique proved its selectivity due to absence of interference from the tablets’ excipients or the plasma components. Also, the HPLC peaks purity angle was within the threshold limits. Overlapped emission spectra of SEMG recorded at time intervals across its peak (Fig. 4) shows the purity of the peaks. Figure 3 also shows blank plasma and plasma spiked with CLZ showing no interference at R_t_ of SEMG.

#### Stability of Spiked Samples

The stability results of SEMG was tested using two of the quality control standards prepared (LQC and HQC) (n = 6) under the conditions stated in Table 5. The RSD% and Er% did not exceed the limits demonstrating stability of the samples under the studied conditions.

**Table 5:** Stability tests in plasma samples (*n* = 6) using the proposed HPLC method

Stability	QC samples	Mean % Recovery ± %RSD	%E_r_
Short-term (25 °C, 6 h)	LQC	102.50 ± 1.30	2.50
	HQC	101.36 ± 1.55	1.36
Freeze–thaw (3 cycles, − 70 °C)	LQC	96.90 ± 1.89	-3.10
	HQC	100.25 ± 1.88	0.25
Post preparative (5 °C for 24 h)	LQC	103.10 ± 1.99	3.10
	HQC	102.90 ± 1.79	2.90
Long-term (45 days, − 70 °C)	LQC	99.99 ± 1.80	-0.01
	HQC	99.70 ± 1.65	-0.30

#### Applications

##### For Tablets Dosage Form Analysis

The HPLC method was implemented for the analysis of SEMG in its tablets. The obtained results shown in Table 6 were in agreement with the labeled claims and the proposed methods were compared with a reported one and no significant difference was noticed comparing both methods.

**Table 6:** Application of the proposed methods for SEMG assay in its tablets dosage forms

Rybelsus® Tablets	% Found ± RSD % *(n* = *5)*	
	Reference method [14]	HPLC- Fluorimetric detection
	100.35 ± 1.18	100.22 ± 0.99
Students’ *t- test* (*t*)^*^	1.16	
Variance ratio F- test (F)^*^	1.35	

^*^Theoretical values of *t* and *F*: 2.31 & 6.39, respectively, at 95% confidence limit

##### For Plasma Samples

As previously discussed,** t**he developed method was applied for SEMG determination in plasma with only protein precipitation and with low LLOD below the reported C_max_ of SEMG.

#### Assessment of Productivity, Greenness and Sustainability

Latterly, it has been agreed that published analytical methodologies should be routinely assessed using different assessment metrics to ensure environmental friendliness, validation, performance and friendly ecological impact. Two of the most recent greenness assessment tools are the “Analytical GREEnness Metric Approach “(AGREE) [19] and the “Complementary green analytical procedure index “(ComplexGAPI) [20]. Also, greenness, sustainability and validation of an analytical procedure can be confirmed by the whiteness assessment done using a new simple in-use algorithm Red–Green–Blue (RGB) 12 model [21].

**Fig. 5:**
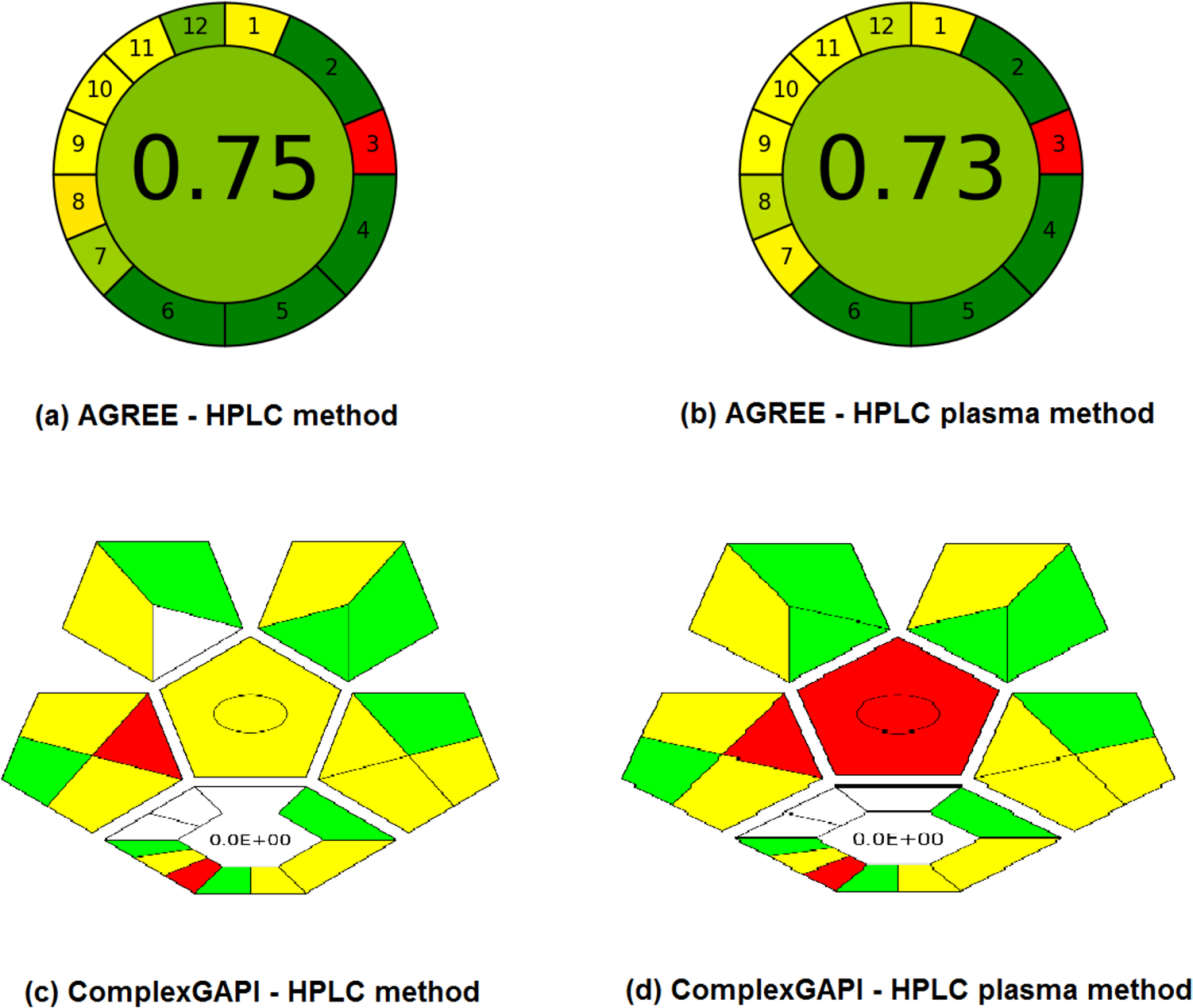
AGREE greenness assessment for (**a**) HPLC method & (**b**) HPLC in plasma method and ComplexGAPI greenness assessment for (**c**) HPLC method & (**d**) HPLC plasma method

The AGREE assesses greenness by using the 12 known green analytical chemistry fundamentals by an easy data entry software and providing the results in the form of a pictogram. From the scores obtained, the HPLC scored 0.75 and the HPLC in spiked plasma scored 0.73 which scored the least due to the extra solvents usage and extraction procedures from the plasma (Figs. 5a & b). Generally both methods are considered green with relatively high scores.

The ComplexGAPI [22] expands its capacity to evaluate a wider array of data, including pre-analysis processes, thus delivering a more thorough assessment of environmental sustainability (Figs. 5c & d). Upon comparing the two diagrams, it becomes apparent that the HPLC method demonstrates fewer red compartments in contrast to the HPLC in plasma method, which is justifiable due to reduced extraction and solvent usage. Furthermore, both methods share a common red compartment for offline analysis. The HPLC method in plasma shows a red pentagram in the middle as a result of the extraction processes needed from plasma samples.

**Fig. 6:**
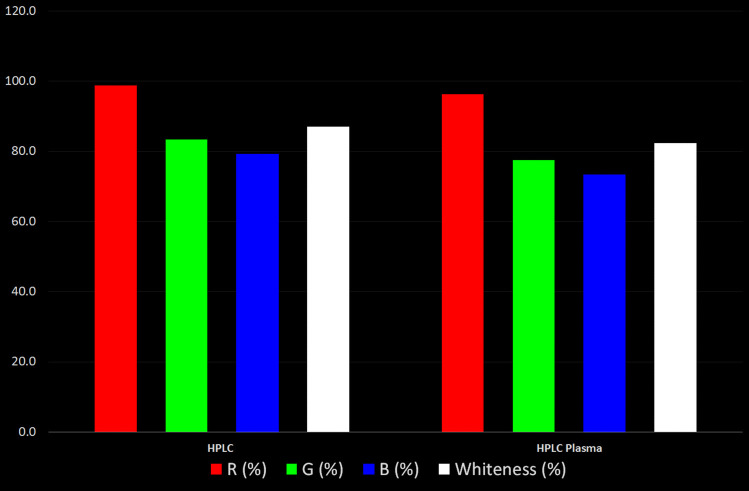
Assessment of both HPLC and HPLC plasma methods using the whiteness RGB 12 model

Red, Green and Blue model is used for whiteness assessment where it is divided into three areas to evaluate the method greenness through the green areas, the validation done on analytical procedures through the red areas and the sustainability as well as productivity through the blue areas as shown in Fig. 6. Results shown in the red area showed that the highest score goes to the HPLC method followed by the HPLC method in plasma, while the HPLC plasma method had the highest sensitivity with the lowest LOQ. The results from the green area did not differ from that of the greenness assessments done using the two previously discussed tools showing harmony in the results. Regarding the blue area, the best score was for the HPLC method followed by the HPLC method in plasma and this is mainly due to the low cost, high speed of analysis and high automation. As a total result from the 3 colors, the HPLC method was with a total whiteness score of 87.1%, and in plasma scored whiteness score 82.4%.

## Conclusion

This work proposes a fluorescence based HPLC method for analysis of SEMG in plasma as it is currently one of the commonly used drugs to monitor diabetes as well as obesity. The HPLC method proposed showed many advantages, the main of which is the sensitivity and selectivity of the method as it combined the high resolution capabilities of chromatography together with the drug’s flourimetric behavior which enabled its determination in low concentration levels. Also, the current HPLC method is eco-friendly and white which was proved by three different scales.

## Supplementary Information

Below is the link to the electronic supplementary material.

Supplementary file1 (DOCX 19 KB)

## Data Availability

No datasets were generated or analysed during the current study.
