# Supplementary material for: RGB Trichromatic Whiteness Assessment of Bio Analytical Chromatographic Tool Using Fluorescence for Quantitation of Semaglutide: Application to Pharmaceutical Preparations and Spiked Plasma
Source: J Fluoresc. 2024 Oct 28;35(8):6467–77. doi: 10.1007/s10895-024-03954-9 (PMC12476385; doi:10.1007/s10895-024-03954-9)
Supplement: Supplementary file 1 [file 10895_2024_3954_MOESM1_ESM.docx]

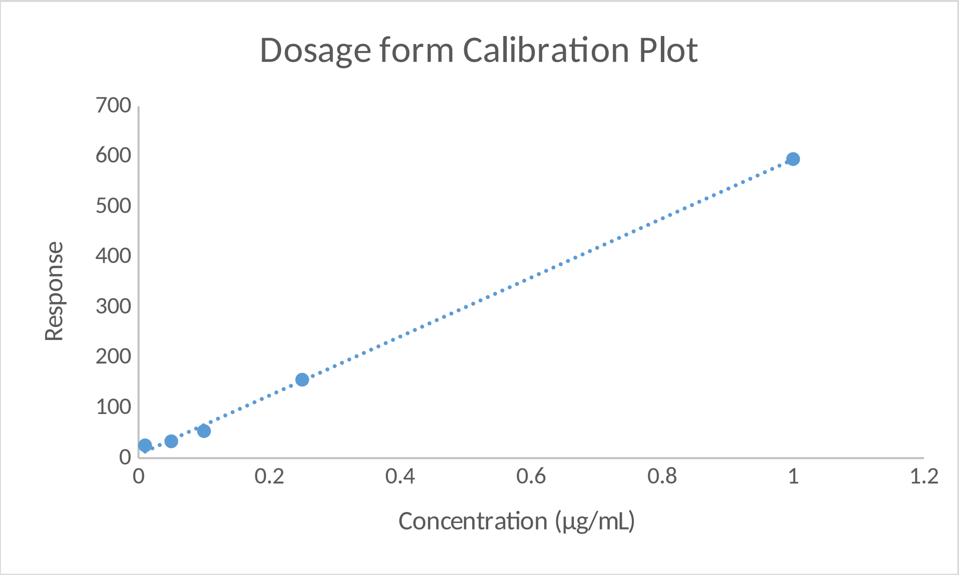

Dosage form Calibration Plot
700
600
500
400
300
200
100
0
Response
0
0.2
0.4
0.6
0.8
1
1.2
Concentration (µg/mL)

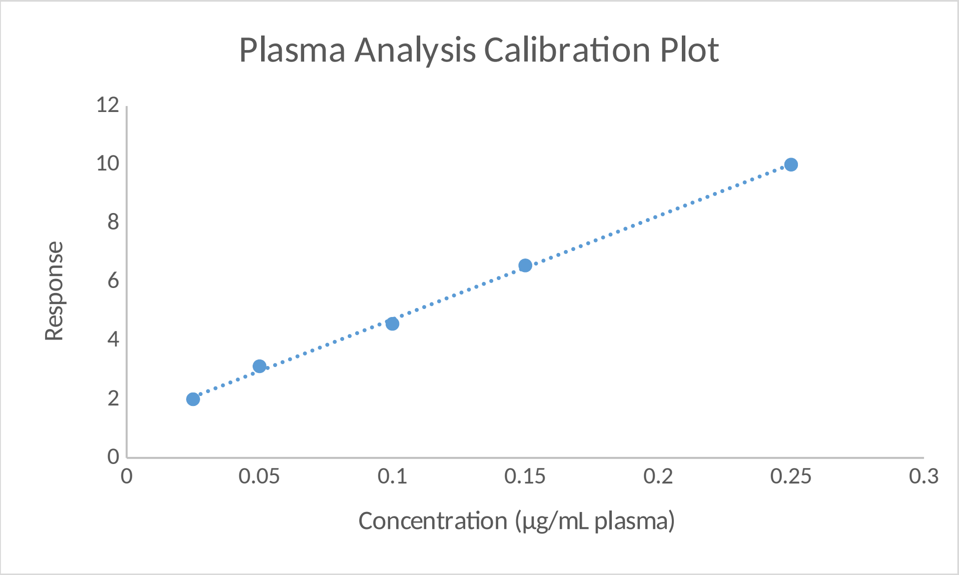

Plasma Analysis Calibration Plot
12
10
8
6
4
2
0
Response
0
0.05
0.1
0.15
0.2
0.25
0.3
Concentration (µg/mL plasma)
